# Synthesis and crystal structure of *catena*-poly[[[aqua­{2-[(*E*)-(1-cyano-2-imino-2-meth­oxy­ethyl­idene)hydrazin­yl]benzene­sulfonato}­sodium]-di-μ-aqua] dihydrate]

**DOI:** 10.1107/S2056989023003602

**Published:** 2023-04-28

**Authors:** Vusala A. Aliyeva, Fargana S. Aliyeva, Mehmet Akkurt, Sema Öztürk Yıldırım, Ajaya Bhattarai

**Affiliations:** a Centro de Química Estrutural, Institute of Molecular Sciences, Instituto Superior Técnico, Universidade de Lisboa, Av. Rovisco Pais, 1049-001 Lisboa, Portugal; b Excellence Center, Baku State University, Z. Xalilov Str. 23, Az 1148 Baku, Azerbaijan; c Department of Physics, Faculty of Sciences, Erciyes University, 38039 Kayseri, Türkiye; d Department of Physics, Faculty of Science, Eskisehir Technical University, Yunus Emre Campus, 26470 Eskisehir, Türkiye; e Department of Chemistry, M.M.A.M.C. (Tribhuvan University), Biratnagar, Nepal; Vienna University of Technology, Austria

**Keywords:** crystal structure, hydrogen bonds, π–π stacking inter­action, chain structure, hydrazone

## Abstract

In the polymeric title compound, {[Na(C_10_H_9_N_4_O_4_S)(H_2_O)_3_]·2H_2_O}_*n*_, sixfold coordinated Na^+^ cations are linked into a chain parallel to [010] by sharing common water mol­ecules. Next to the four bridging water mol­ecules, each Na^+^ cation of the chain is bonded to the O atom of a terminal water mol­ecule and an O atom of the SO_3_^−^ group of the sulfonate anion. Classical O—H⋯O, O—H⋯N and N—H⋯O hydrogen bonds and additional π–π inter­actions connect these chains into a three-dimensional network.

## Abstract 2

In the crystal struture of the title compound, Na^+^ cations form a chain parallel to [010] through bonding to common water mol­ecules.

## 1. Chemical context

Hydrazones are inter­esting compounds in the fields of coordination chemistry, crystal engineering, catalysis, mol­ecular recoginition and synthetic organic chemistry (Ma *et al.*, 2021 ▸; Mahmoudi *et al.*, 2017*a* ▸,*b* ▸, 2019 ▸). Depending on the attached functional groups of the hydrazone ligands, the solubility and catalytic activities of their corresponding metal complexes can be improved (Gurbanov *et al.*, 2022 ▸). Hydrazones have been applied as analytical reagents (Mahmudov *et al.*, 2010 ▸), as well as building blocks in the construction of supra­molecular networks, owing to their capabilities as donor and acceptor groups in hydrogen bonding (Maharramov *et al.*, 2010 ▸; Mahmudov *et al.*, 2011 ▸, 2012 ▸, 2013 ▸). Both resonance-assisted hydrogen or chalcogen bonds also play a crucial role in the synthesis and structural chemistry of hydrazone ligands (Gurbanov *et al.*, 2020*a* ▸,*b* ▸; Mahmudov *et al.*, 2022 ▸). Similar to other classes of N-containing ligands, hydrazones also participate in various types of inter­molecular inter­actions (Polyanskii *et al.*, 2019 ▸; Zubkov *et al.*, 2018 ▸). 
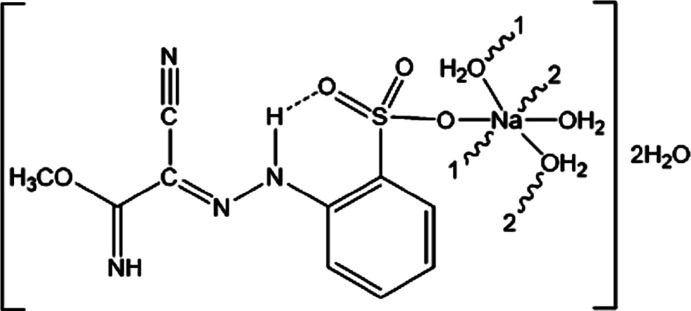


Herein, we report the structural features of the hydrazone derivative poly[[di-μ-aqua-{2-[(*E*)-(1-cyano-2-imino-2-meth­oxy­ethyl­idene)hydrazin­yl]benzene­sulfonato}­sodium] dihydrate].

## 2. Structural commentary

**Figure 1:**
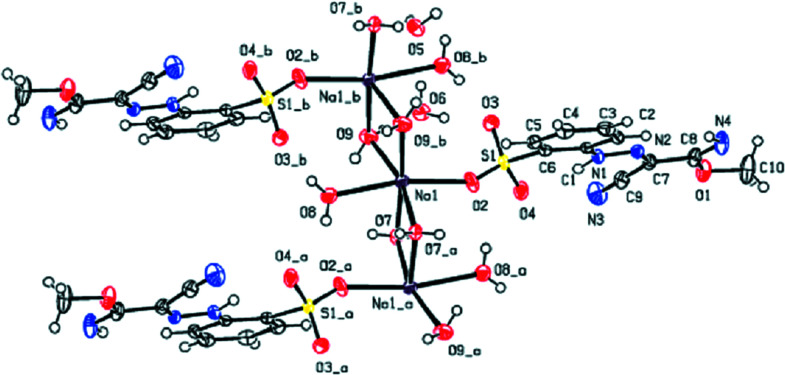
The coordination environment of the Na^+^ cations in the title compound, leading to the formation of a chain extending parallel to [010]. Displacement ellipsoids are drawn at the 50% probability level. [Symmetry codes: (*a*) −*x*, −*y*, −*z* + 1; (*b*) −*x*, −*y* + 1, −*z* + 1.]

The Na^+^ cation exhibits a six-coordination by O atoms in the form of a distorted octa­hedron. Four water mol­ecules (O7, O9 and their symmetry-related counterparts) bridge adjacent cations into a chain extending parallel to [010] (Fig. 1 ▸). The coordination sphere is completed by the O atoms of another water mol­ecule (O8), now bonded terminally, and an O atom from the SO_3_^−^ group (O2). The sulfonate anion shows no atypical features.

## 3. Supra­molecular features

**Figure 2:**
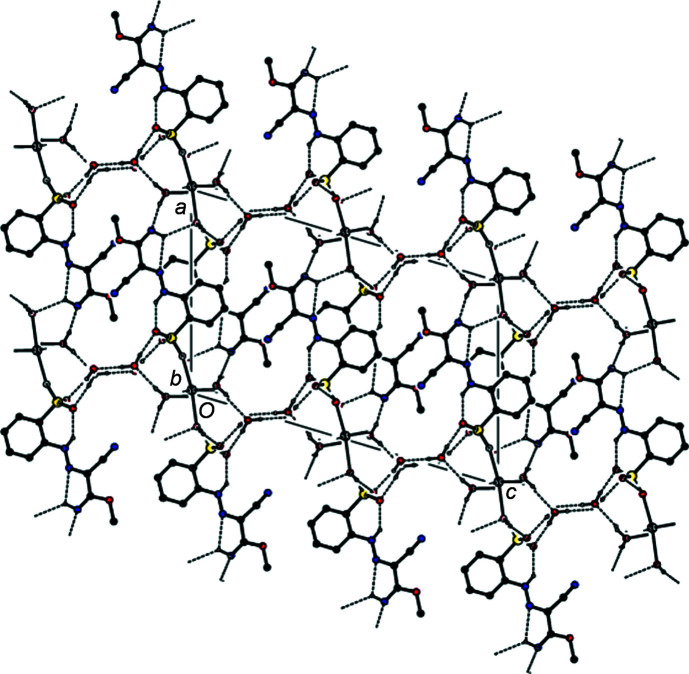
View of the crystal structure along [010], with O—H⋯O, O—H⋯N and N—H⋯O hydrogen bonds shown as dashed lines.

**Figure 4:**
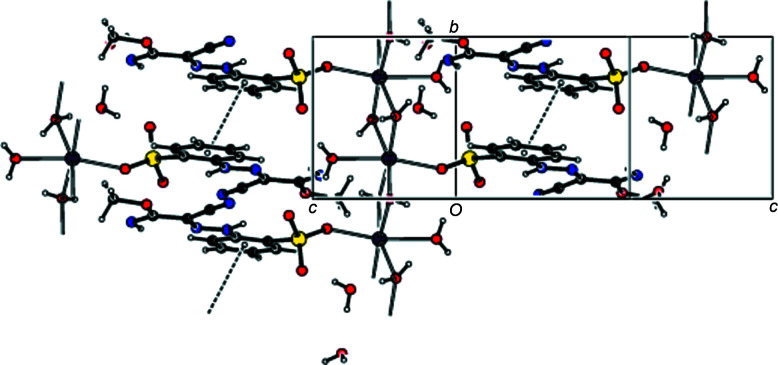
The π–π stacking inter­actions in the title compound shown with dashed lines.

Classical O—H⋯O, O—H⋯N and N—H⋯O hydrogen bonds of medium strength (Table 1 ▸) form a three-dimensional network (Figs. 2 ▸ and 3 ▸). Furthermore, the mol­ecules are linked by π–π stacking inter­actions between the benzene rings [*Cg*1⋯*Cg*1(−*x* + 1, −*y* + 1, −*z* + 1) = 3.7588 (8) Å and slippage = 1.684 Å, where *Cg*1 is the centroid of the C1–C6 ring] parallel to [010] (Fig. 4 ▸).

## 4. Database survey

A search in the Cambridge Structural Database (CSD, Version 5.42, September 2021 update; Groom *et al.*, 2016 ▸) for related benzene­sulfonates with a monovalent cation gave two matches. In *catena*-[bis­(μ_4_-3-carb­oxy-4-hy­droxy­benzene­sul­fon­ato)­tri­aqua­disilver(I) monohydrate] (CSD refcode FETHES; Gao *et al.*, 2005 ▸), both substituted benzene­sulfonate anions use two of their sulfonyl O atoms to link to three Ag^+^ cations and their carbonyl O atom to another Ag^+^ cation in a μ_4_-binding mode. The two symmetry-independent Ag^+^ cations are additionally coordinated by water mol­ecules, one by one water mol­ecule and the other by two water mol­ecules, so that one Ag^+^ cation is five- and the other six-coordinate. In *catena*-[μ_5_-(3-carb­oxy-4-hy­droxy­benzene­sulfonate)(μ_2_-aqua)­ru­bidium] (FAXGAN; Hu *et al.*, 2005 ▸), the 3-carb­oxy-4-hy­droxy­ben­zene­sulfonate anion retains the usual inter­molecular hydrogen bond between the phenol and carboxyl O atoms. The Rb^+^ cation is surrounded by eight O atoms, and the crystal packing is stabilized by inter­molecular O—H⋯O hydrogen bonds.

## 5. Synthesis and crystallization

344 mg (1 mmol) of sodium 2-[2-(di­cyano­methyl­ene)hy­dra­zin­yl]benzene­sulfonate tetra­hydrate (Kopylovich *et al.*, 2013 ▸) were dissolved in 60 ml of methanol and refluxed for 6 h. The reaction mixture was kept in air at room temperature for slow evaporation. After *ca* 2–3 d, yellow crystals of the title compound, suitable for X-ray analysis, had formed (yield 84%). The crystals were soluble in DMSO, ethanol and di­methyl­formamide and insoluble in nonpolar solvents. Elemental analysis (%) for C_10_H_19_N_4_NaO_9_S: C 30.41 (calc. 30.46), H 4.83 (4.86), N 14.16 (14.21); IR (KBr): 3390 ν(OH), 2995 and 2867 ν(NH), 1653 ν(C=N) cm^−1^. ^1^H NMR in DMSO, inter­nal TMS: δ (ppm) 3.44 (3H, OCH_3_), 7.16–8.11 (4H, Ar—H), 10.19 (1H, NH), 14.11 (*s*, 1H, N—H). ^13^C NMR in DMSO, inter­nal TMS: δ (ppm) 58.2 (OCH_3_), 111.5 (C=N), 112.3 (C≡N), 123.6 (ArC—H), 121.7 (ArC—SO_3_Na), 122.2 (ArC—H), 126.8 (ArC—H), 129.1 (ArC—H), 142.5 (ArC—NH) and 160.0 (C=NH).

## 6. Refinement

Crystal data, data collection and structure refinement details are summarized in Table 2 ▸. C-bound H atoms were positioned geometrically (C—H = 0.95 and 0.98 Å) and refined using a riding model, with *U*_iso_(H) = 1.2 or 1.5*U*_eq_(C). O- and N-bound H atoms were located from difference Fourier maps and refined with *U*_iso_(H) = 1.2*U*_eq_(N) and 1.5*U*_eq_(O), with their positions fixed at distances of N—H = 0.93 Å and O—H = 0.85 Å.

## Supplementary Material

Crystal structure: contains datablock(s) I, global. DOI: 10.1107/S2056989023003602/wm5680sup1.cif

Structure factors: contains datablock(s) I. DOI: 10.1107/S2056989023003602/wm5680Isup2.hkl

CCDC reference: 2257827

Additional supporting information:  crystallographic information; 3D view; checkCIF report

## Figures and Tables

**Figure 3:**
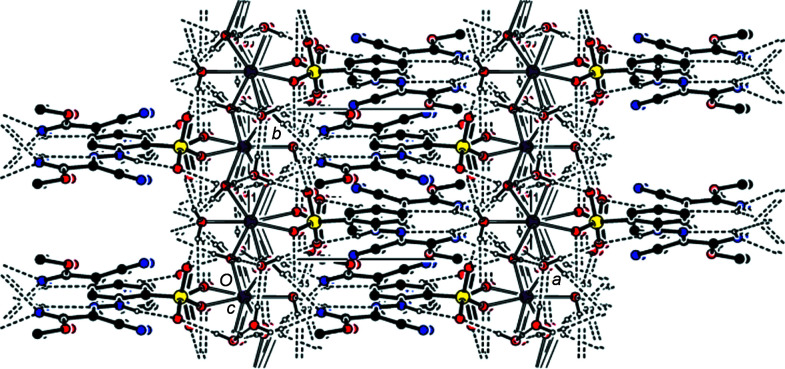
View of the crystal structure along [001]; the hydrogen bonds are as in Fig. 2 ▸.

**Table 1:** Hydrogen-bond geometry (Å, °)

*D*—H⋯*A*	*D*—H	H⋯*A*	*D*⋯*A*	*D*—H⋯*A*
O5—H5*A*⋯O4^i^	0.85	1.98	2.8243 (13)	173
O5—H5*B*⋯O6^ii^	0.85	1.92	2.7660 (13)	176
O6—H6*A*⋯O5	0.85	1.88	2.7295 (15)	174
O6—H6*B*⋯O3	0.85	1.98	2.8169 (13)	168
O7—H7*A*⋯O5^iii^	0.85	2.06	2.8978 (13)	171
O7—H7*B*⋯N4^iv^	0.85	1.98	2.8141 (14)	167
O8—H8*A*⋯O3^v^	0.85	2.05	2.8874 (14)	168
O8—H8*B*⋯O2^vi^	0.85	2.18	2.9689 (15)	153
O9—H9*A*⋯O6	0.85	1.97	2.8241 (14)	178
N4—H4N⋯O8^vii^	0.93	2.38	3.0905 (16)	133

Symmetry codes: (i) 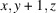 ; (ii) 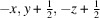 ; (iii) 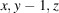 ; (iv) 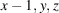 ; (v) 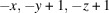 ; (vi) 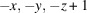 ; (vii) 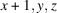 .

**Table 2:** Experimental details

Crystal data	
Chemical formula	[Na(C_10_H_9_N_4_O_4_S)(H_2_O)_3_]·2H_2_O
*M*_r_	394.34
Crystal system, space group	Monoclinic, *P*2_1_/*c*
Temperature (K)	150
*a*, *b*, *c* (Å)	13.3305 (5), 6.8212 (3), 20.9547 (8)
β (°)	106.681 (1)
*V* (Å^3^)	1825.23 (13)
*Z*	4
Radiation type	Mo *K*α
μ (mm^−1^)	0.25
Crystal size (mm)	0.27 × 0.21 × 0.10

Data collection	
Diffractometer	Bruker APEXII CCD
Absorption correction	Multi-scan (*SADABS*; Krause *et al.*, 2015 ▸)
*T*_min_, *T*_max_	0.926, 0.967
No. of measured, independent and observed [*I* > 2σ(*I*)] reflections	27710, 3748, 3389
*R*_int_	0.021
(sin θ/λ)_max_ (Å^−1^)	0.628

Refinement	
*R*[*F* ^2^ > 2σ(*F* ^2^)], *wR*(*F* ^2^), *S*	0.028, 0.081, 1.07
No. of reflections	3748
No. of parameters	227
H-atom treatment	H-atom parameters constrained
Δρ_max_, Δρ_min_ (e Å^−3^)	0.35, −0.36

Computer programs: *APEX2* and *SAINT* (Bruker, 2007 ▸), *SHELXS* (Sheldrick, 2008 ▸), *SHELXL2019* (Sheldrick, 2015 ▸), *ORTEP-3 for Windows* (Farrugia, 2012 ▸) and *PLATON* (Spek, 2020 ▸).

## supplementary crystallographic information

### Crystal data

**Table:**

[Na(C_10_H_9_N_4_O_4_S)(H_2_O)_3_]·2H_2_O	*F*(000) = 824
*M**_r_* = 394.34	*D*_x_ = 1.435 Mg m^−^^3^
Monoclinic, *P*2_1_/*c*	Mo *K*α radiation, λ = 0.71073 Å
*a* = 13.3305 (5) Å	Cell parameters from 9914 reflections
*b* = 6.8212 (3) Å	θ = 3.2–26.5°
*c* = 20.9547 (8) Å	µ = 0.25 mm^−^^1^
β = 106.681 (1)°	*T* = 150 K
*V* = 1825.23 (13) Å^3^	Needle, yellow
*Z* = 4	0.27 × 0.21 × 0.10 mm

### Data collection

**Table:**

Bruker APEXII CCD diffractometer	3389 reflections with *I* > 2σ(*I*)
φ and ω scans	*R*_int_ = 0.021
Absorption correction: multi-scan (*SADABS*; Krause *et al.*, 2015)	θ_max_ = 26.5°, θ_min_ = 2.2°
*T*_min_ = 0.926, *T*_max_ = 0.967	*h* = −16→16
27710 measured reflections	*k* = −8→7
3748 independent reflections	*l* = −26→26

### Refinement

**Table:**

Refinement on *F*^2^	Primary atom site location: structure-invariant direct methods
Least-squares matrix: full	Secondary atom site location: difference Fourier map
*R*[*F*^2^ > 2σ(*F*^2^)] = 0.028	Hydrogen site location: mixed
*wR*(*F*^2^) = 0.081	H-atom parameters constrained
*S* = 1.07	*w* = 1/[σ^2^(*F*_o_^2^) + (0.0397*P*)^2^ + 0.8377*P*] where *P* = (*F*_o_^2^ + 2*F*_c_^2^)/3
3748 reflections	(Δ/σ)_max_ = 0.001
227 parameters	Δρ_max_ = 0.35 e Å^−^^3^
0 restraints	Δρ_min_ = −0.36 e Å^−^^3^

### Special details

**Table:**

Geometry. All esds (except the esd in the dihedral angle between two l.s. planes) are estimated using the full covariance matrix. The cell esds are taken into account individually in the estimation of esds in distances, angles and torsion angles; correlations between esds in cell parameters are only used when they are defined by crystal symmetry. An approximate (isotropic) treatment of cell esds is used for estimating esds involving l.s. planes.

### Fractional atomic coordinates and isotropic or equivalent isotropic displacement parameters (Å^2^)

**Table:**

	*x*	*y*	*z*	*U*_iso_*/*U*_eq_	
Na1	0.01633 (4)	0.24885 (7)	0.50226 (3)	0.02094 (13)	
S1	0.24044 (2)	0.24765 (4)	0.43523 (2)	0.01763 (9)	
O1	0.64543 (8)	0.03548 (17)	0.26660 (5)	0.0316 (2)	
O2	0.16843 (7)	0.18478 (17)	0.47128 (5)	0.0312 (2)	
O3	0.21772 (7)	0.44290 (14)	0.40648 (5)	0.0250 (2)	
O4	0.24943 (7)	0.10353 (15)	0.38547 (5)	0.0292 (2)	
O5	0.05048 (7)	0.95638 (15)	0.31489 (4)	0.0259 (2)	
H5A	0.111073	1.003570	0.332701	0.039*	
H5B	0.026423	0.994300	0.274741	0.039*	
O6	0.02512 (8)	0.55977 (15)	0.31795 (5)	0.0278 (2)	
H6A	0.036537	0.682516	0.319429	0.042*	
H6B	0.083707	0.512606	0.340269	0.042*	
O7	−0.05415 (7)	0.00275 (14)	0.41756 (4)	0.0214 (2)	
H7A	−0.029422	−0.003553	0.384492	0.032*	
H7B	−0.116822	0.035287	0.397482	0.032*	
O8	−0.15677 (8)	0.24814 (14)	0.51810 (5)	0.0267 (2)	
H8A	−0.183026	0.329914	0.539397	0.040*	
H8B	−0.170926	0.135404	0.530527	0.040*	
O9	−0.07335 (8)	0.48983 (15)	0.41848 (5)	0.0292 (2)	
H9A	−0.044937	0.508569	0.387489	0.044*	
H9B	−0.120247	0.402548	0.405278	0.044*	
N1	0.45695 (8)	0.19223 (16)	0.41179 (5)	0.0173 (2)	
H1N	0.394133	0.153936	0.384683	0.021*	
N2	0.54648 (8)	0.17969 (16)	0.39713 (5)	0.0184 (2)	
N3	0.37653 (11)	0.0270 (2)	0.24994 (7)	0.0407 (3)	
N4	0.73936 (9)	0.1439 (2)	0.37031 (6)	0.0331 (3)	
H4N	0.728594	0.183478	0.410070	0.040*	
C1	0.45890 (9)	0.23787 (17)	0.47751 (6)	0.0160 (2)	
C2	0.55436 (10)	0.25797 (18)	0.52682 (7)	0.0203 (3)	
H2	0.618229	0.240330	0.515954	0.024*	
C3	0.55603 (11)	0.3035 (2)	0.59141 (6)	0.0243 (3)	
H3	0.621321	0.316534	0.624649	0.029*	
C4	0.46382 (11)	0.3304 (2)	0.60838 (6)	0.0254 (3)	
H4	0.465840	0.363154	0.652795	0.030*	
C5	0.36862 (10)	0.30915 (19)	0.55994 (6)	0.0220 (3)	
H5	0.305188	0.326402	0.571371	0.026*	
C6	0.36531 (9)	0.26271 (17)	0.49470 (6)	0.0167 (2)	
C7	0.54846 (10)	0.12221 (19)	0.33855 (6)	0.0206 (3)	
C8	0.65335 (10)	0.1044 (2)	0.32786 (6)	0.0254 (3)	
C9	0.45515 (11)	0.0676 (2)	0.28728 (6)	0.0253 (3)	
C10	0.74403 (13)	0.0023 (3)	0.25222 (8)	0.0457 (5)	
H10C	0.730573	−0.048535	0.206797	0.069*	
H10D	0.785594	−0.093168	0.283906	0.069*	
H10E	0.782662	0.126071	0.256239	0.069*	

### Atomic displacement parameters (Å^2^)

**Table:**

	*U*^11^	*U*^22^	*U*^33^	*U*^12^	*U*^13^	*U*^23^
Na1	0.0226 (3)	0.0185 (3)	0.0246 (3)	0.00087 (19)	0.0112 (2)	0.00084 (19)
S1	0.01257 (15)	0.01943 (17)	0.02187 (16)	−0.00042 (10)	0.00653 (12)	0.00011 (11)
O1	0.0268 (5)	0.0499 (7)	0.0209 (5)	0.0097 (5)	0.0111 (4)	−0.0017 (4)
O2	0.0193 (5)	0.0428 (6)	0.0354 (5)	−0.0046 (4)	0.0140 (4)	0.0061 (5)
O3	0.0219 (4)	0.0237 (5)	0.0267 (5)	0.0024 (4)	0.0027 (4)	0.0043 (4)
O4	0.0163 (4)	0.0326 (5)	0.0361 (5)	−0.0006 (4)	0.0034 (4)	−0.0151 (4)
O5	0.0229 (4)	0.0335 (5)	0.0192 (4)	−0.0052 (4)	0.0025 (4)	0.0012 (4)
O6	0.0286 (5)	0.0276 (5)	0.0229 (5)	0.0014 (4)	0.0007 (4)	0.0009 (4)
O7	0.0172 (4)	0.0256 (5)	0.0218 (4)	0.0030 (3)	0.0063 (3)	0.0015 (4)
O8	0.0287 (5)	0.0237 (5)	0.0316 (5)	0.0012 (4)	0.0147 (4)	−0.0001 (4)
O9	0.0352 (5)	0.0240 (5)	0.0305 (5)	−0.0043 (4)	0.0126 (4)	0.0006 (4)
N1	0.0139 (5)	0.0202 (5)	0.0183 (5)	−0.0010 (4)	0.0052 (4)	−0.0009 (4)
N2	0.0170 (5)	0.0173 (5)	0.0226 (5)	0.0020 (4)	0.0085 (4)	0.0034 (4)
N3	0.0338 (7)	0.0553 (9)	0.0308 (7)	−0.0053 (6)	0.0059 (6)	−0.0077 (6)
N4	0.0215 (6)	0.0581 (9)	0.0214 (5)	0.0079 (6)	0.0088 (5)	0.0006 (6)
C1	0.0175 (6)	0.0125 (5)	0.0180 (6)	−0.0012 (4)	0.0053 (5)	0.0013 (4)
C2	0.0172 (6)	0.0191 (6)	0.0233 (6)	−0.0015 (4)	0.0038 (5)	0.0011 (5)
C3	0.0259 (6)	0.0215 (6)	0.0211 (6)	−0.0039 (5)	−0.0005 (5)	0.0009 (5)
C4	0.0372 (7)	0.0224 (7)	0.0165 (6)	−0.0018 (6)	0.0076 (5)	−0.0009 (5)
C5	0.0270 (6)	0.0195 (6)	0.0230 (6)	0.0003 (5)	0.0125 (5)	0.0008 (5)
C6	0.0169 (6)	0.0141 (6)	0.0194 (6)	−0.0003 (4)	0.0059 (5)	0.0014 (4)
C7	0.0208 (6)	0.0220 (6)	0.0202 (6)	0.0030 (5)	0.0079 (5)	0.0028 (5)
C8	0.0237 (6)	0.0334 (7)	0.0220 (6)	0.0083 (5)	0.0111 (5)	0.0053 (5)
C9	0.0275 (7)	0.0299 (7)	0.0214 (6)	0.0019 (6)	0.0118 (5)	−0.0008 (5)
C10	0.0317 (8)	0.0826 (14)	0.0273 (7)	0.0189 (8)	0.0155 (6)	−0.0014 (8)

### Geometric parameters (Å, º)

**Table:**

Na1—O2	2.3407 (11)	O9—H9B	0.8500
Na1—O7^i^	2.3530 (10)	N1—N2	1.3178 (14)
Na1—O9^ii^	2.4061 (11)	N1—C1	1.4049 (15)
Na1—O8	2.4237 (11)	N1—H1N	0.9050
Na1—O7	2.4276 (10)	N2—C7	1.2961 (16)
Na1—O9	2.4511 (11)	N3—C9	1.1474 (19)
Na1—Na1^i^	3.4206 (10)	N4—C8	1.2621 (18)
Na1—Na1^ii^	3.4517 (10)	N4—H4N	0.9254
Na1—H9B	2.5336	C1—C2	1.3967 (17)
S1—O2	1.4466 (9)	C1—C6	1.4045 (17)
S1—O3	1.4572 (10)	C2—C3	1.3826 (19)
S1—O4	1.4627 (10)	C2—H2	0.9500
S1—C6	1.7730 (13)	C3—C4	1.386 (2)
O1—C8	1.3424 (16)	C3—H3	0.9500
O1—C10	1.4482 (17)	C4—C5	1.3864 (19)
O5—H5A	0.8498	C4—H4	0.9500
O5—H5B	0.8501	C5—C6	1.3916 (18)
O6—H6A	0.8500	C5—H5	0.9500
O6—H6B	0.8500	C7—C9	1.4398 (18)
O7—H7A	0.8496	C7—C8	1.4832 (17)
O7—H7B	0.8500	C10—H10C	0.9800
O8—H8A	0.8500	C10—H10D	0.9800
O8—H8B	0.8503	C10—H10E	0.9800
O9—H9A	0.8501		
			
O2—Na1—O7^i^	92.10 (4)	Na1^i^—O7—H7B	121.5
O2—Na1—O9^ii^	101.69 (4)	Na1—O7—H7B	107.5
O7^i^—Na1—O9^ii^	94.98 (4)	H7A—O7—H7B	99.5
O2—Na1—O8	166.44 (4)	Na1—O8—H8A	128.5
O7^i^—Na1—O8	85.30 (3)	Na1—O8—H8B	110.3
O9^ii^—Na1—O8	91.80 (4)	H8A—O8—H8B	105.8
O2—Na1—O7	81.51 (4)	Na1^ii^—O9—Na1	90.57 (4)
O7^i^—Na1—O7	88.64 (3)	Na1^ii^—O9—H9A	107.1
O9^ii^—Na1—O7	175.04 (4)	Na1—O9—H9A	114.5
O8—Na1—O7	85.12 (3)	Na1^ii^—O9—H9B	143.2
O2—Na1—O9	102.11 (4)	Na1—O9—H9B	85.7
O7^i^—Na1—O9	163.96 (4)	H9A—O9—H9B	107.7
O9^ii^—Na1—O9	89.43 (4)	N2—N1—C1	118.67 (10)
O8—Na1—O9	79.14 (4)	N2—N1—H1N	124.9
O7—Na1—O9	86.18 (4)	C1—N1—H1N	115.6
O2—Na1—Na1^i^	85.44 (3)	C7—N2—N1	120.42 (11)
O7^i^—Na1—Na1^i^	45.20 (3)	C8—N4—H4N	110.6
O9^ii^—Na1—Na1^i^	140.07 (4)	C2—C1—C6	119.09 (11)
O8—Na1—Na1^i^	83.29 (3)	C2—C1—N1	120.22 (11)
O7—Na1—Na1^i^	43.45 (2)	C6—C1—N1	120.68 (11)
O9—Na1—Na1^i^	127.86 (4)	C3—C2—C1	120.10 (12)
O2—Na1—Na1^ii^	106.87 (4)	C3—C2—H2	120.0
O7^i^—Na1—Na1^ii^	137.99 (4)	C1—C2—H2	120.0
O9^ii^—Na1—Na1^ii^	45.24 (3)	C2—C3—C4	120.96 (12)
O8—Na1—Na1^ii^	83.58 (3)	C2—C3—H3	119.5
O7—Na1—Na1^ii^	130.32 (3)	C4—C3—H3	119.5
O9—Na1—Na1^ii^	44.19 (3)	C3—C4—C5	119.41 (12)
Na1^i^—Na1—Na1^ii^	166.02 (3)	C3—C4—H4	120.3
O2—Na1—H9B	109.6	C5—C4—H4	120.3
O7^i^—Na1—H9B	146.3	C4—C5—C6	120.48 (12)
O9^ii^—Na1—H9B	105.0	C4—C5—H5	119.8
O8—Na1—H9B	67.5	C6—C5—H5	119.8
O7—Na1—H9B	70.2	C5—C6—C1	119.95 (11)
O9—Na1—H9B	19.5	C5—C6—S1	117.54 (10)
Na1^i^—Na1—H9B	109.4	C1—C6—S1	122.45 (9)
Na1^ii^—Na1—H9B	60.7	N2—C7—C9	122.47 (11)
O2—S1—O3	113.36 (6)	N2—C7—C8	116.37 (11)
O2—S1—O4	112.05 (6)	C9—C7—C8	121.07 (11)
O3—S1—O4	111.68 (6)	N4—C8—O1	123.70 (12)
O2—S1—C6	106.26 (6)	N4—C8—C7	125.53 (12)
O3—S1—C6	106.22 (5)	O1—C8—C7	110.75 (11)
O4—S1—C6	106.71 (5)	N3—C9—C7	174.65 (14)
C8—O1—C10	115.26 (11)	O1—C10—H10C	109.5
S1—O2—Na1	147.89 (7)	O1—C10—H10D	109.5
H5A—O5—H5B	111.1	H10C—C10—H10D	109.5
H6A—O6—H6B	103.1	O1—C10—H10E	109.5
Na1^i^—O7—Na1	91.36 (3)	H10C—C10—H10E	109.5
Na1^i^—O7—H7A	119.5	H10D—C10—H10E	109.5
Na1—O7—H7A	118.2		
			
O3—S1—O2—Na1	5.69 (15)	N1—C1—C6—S1	−2.39 (16)
O4—S1—O2—Na1	−121.84 (12)	O2—S1—C6—C5	−30.12 (12)
C6—S1—O2—Na1	121.98 (12)	O3—S1—C6—C5	90.88 (10)
C1—N1—N2—C7	174.30 (11)	O4—S1—C6—C5	−149.84 (10)
N2—N1—C1—C2	−3.68 (17)	O2—S1—C6—C1	152.62 (10)
N2—N1—C1—C6	176.71 (11)	O3—S1—C6—C1	−86.38 (11)
C6—C1—C2—C3	−0.59 (18)	O4—S1—C6—C1	32.90 (12)
N1—C1—C2—C3	179.80 (11)	N1—N2—C7—C9	−0.71 (19)
C1—C2—C3—C4	−0.2 (2)	N1—N2—C7—C8	−177.34 (11)
C2—C3—C4—C5	0.7 (2)	C10—O1—C8—N4	1.9 (2)
C3—C4—C5—C6	−0.5 (2)	C10—O1—C8—C7	−176.78 (13)
C4—C5—C6—C1	−0.26 (19)	N2—C7—C8—N4	−1.5 (2)
C4—C5—C6—S1	−177.60 (10)	C9—C7—C8—N4	−178.15 (15)
C2—C1—C6—C5	0.81 (17)	N2—C7—C8—O1	177.19 (12)
N1—C1—C6—C5	−179.58 (11)	C9—C7—C8—O1	0.51 (18)
C2—C1—C6—S1	178.00 (9)		

Symmetry codes: (i) −*x*, −*y*, −*z*+1; (ii) −*x*, −*y*+1, −*z*+1.

### Hydrogen-bond geometry (Å, º)

**Table:**

*D*—H···*A*	*D*—H	H···*A*	*D*···*A*	*D*—H···*A*
O5—H5*A*···O4^iii^	0.85	1.98	2.8243 (13)	173
O5—H5*B*···O6^iv^	0.85	1.92	2.7660 (13)	176
O6—H6*A*···O5	0.85	1.88	2.7295 (15)	174
O6—H6*B*···O3	0.85	1.98	2.8169 (13)	168
O7—H7*A*···O5^v^	0.85	2.06	2.8978 (13)	171
O7—H7*B*···N4^vi^	0.85	1.98	2.8141 (14)	167
O8—H8*A*···O3^ii^	0.85	2.05	2.8874 (14)	168
O8—H8*B*···O2^i^	0.85	2.18	2.9689 (15)	153
O9—H9*A*···O6	0.85	1.97	2.8241 (14)	178
N4—H4*N*···O8^vii^	0.93	2.38	3.0905 (16)	133

Symmetry codes: (i) −*x*, −*y*, −*z*+1; (ii) −*x*, −*y*+1, −*z*+1; (iii) *x*, *y*+1, *z*; (iv) −*x*, *y*+1/2, −*z*+1/2; (v) *x*, *y*−1, *z*; (vi) *x*−1, *y*, *z*; (vii) *x*+1, *y*, *z*.
